# Phenotype prediction and characterization of 25 pharmacogenes in Thais from whole genome sequencing for clinical implementation

**DOI:** 10.1038/s41598-020-76085-3

**Published:** 2020-11-03

**Authors:** John Mauleekoonphairoj, Monpat Chamnanphon, Apichai Khongphatthanayothin, Boosamas Sutjaporn, Pharawee Wandee, Yong Poovorawan, Koonlawee Nademanee, Monnat Pongpanich, Pajaree Chariyavilaskul

**Affiliations:** 1 Department of Medicine, Faculty of Medicine, Center of Excellence in Arrhythmia Research Chulalongkorn University, Chulalongkorn University, Bangkok, Thailand; 2 Interdisciplinary Program of Biomedical Sciences, Graduate School, Chulalongkorn University, Bangkok, Thailand; 3 Clinical Pharmacokinetics and Pharmacogenomics Research Unit, Faculty of Medicine, Chulalongkorn University, Bangkok, Thailand; 4 Department of Pharmacology, Faculty of Medicine, Chulalongkorn University, Bangkok, Thailand; 5 Division of Cardiology, Department of Pediatrics, Faculty of Medicine, Chulalongkorn University, Bangkok, Thailand; 6 Bangkok General Hospital, Bangkok, Thailand; 7 Department of Pediatrics, Faculty of Medicine, Chulalongkorn University, Bangkok, Thailand; 8 Department of Medicine, Faculty of Medicine, Chulalongkorn University, Bangkok, Thailand; 9 Pacific Rim Electrophysiology Research Institute, Bumrungrad Hospital, Bangkok, Thailand; 10 Department of Mathematics and Computer Science, Faculty of Science, Chulalongkorn University, Bangkok, Thailand; 11 Faculty of Science, Omics Sciences and Bioinfomatics Center, Chulalongkorn University, Bangkok, Thailand

**Keywords:** Genomics, Pharmacogenomics, Haplotypes

## Abstract

Publicly available pharmacogenomics (PGx) databases enable translation of genotype data into clinically actionable information. As variation within pharmacogenes is population-specific, this study investigated the spectrum of 25 clinically relevant pharmacogenes in the Thai population (n = 291) from whole genome sequencing. The bioinformatics tool Stargazer was used for phenotype prediction, through assignment of alleles and detection of structural variation. Known and unreported potentially deleterious PGx variants were identified. Over 25% of Thais carried a high-risk diplotype in *CYP3A5*, *CYP2C19*, *CYP2D6*, *NAT2*, *SLCO1B1,* and *UGT1A1. CYP2D6* structural variants accounted for 83.8% of all high-risk diplotypes. Of 39 known PGx variants identified, six variants associated with adverse drug reactions were common. Allele frequencies of CYP3A5*3 (rs776746), CYP2B6*6 (rs2279343), and *NAT2* (rs1041983*)* were significantly higher in Thais than East-Asian and global populations. 121 unreported variants had potential to exert clinical impact, majority were rare and population-specific, with 60.3% of variants absent from gnomAD database. This study demonstrates the population-specific variation in clinically relevant pharmacogenes, the importance of *CYP2D6* structural variation detection in the Thai population, and potential of unreported variants in explaining drug response. These findings are essential in development of dosing guidelines, PGx testing, clinical trials, and drugs.

## Introduction

Implementing pharmacogenomics (PGx) findings into the clinical setting has been a challenge. Initiatives including The Clinical Pharmacogenomics Implementation Consortium (CPIC) and the Pharmacogenomics Knowledge Base (PharmGKB), which gathered evidence-based, peer-reviewed research and treatment recommendations of pharmacogenes, were developed for efficient extraction and translation of genetic information into clinical action^1,2^.

The “star” nomenclature system, commonly use in treatment guidelines, involved identification of alleles, diplotypes and complex structural variation (SV) for accurate phenotype prediction. Accurately assign alleles has been a challenge as tests designed by different laboratory examined different combination of variants resulted in incorrect allele assignments, hence phenotype prediction^3^. This create disparities in reports of the same sample that discourage the adaptation of PGx testing. Whole genome sequencing (WGS) has advantages over other platforms as it identifies all variants required for accurate allele assignment and novel clinically relevant PGx variants, which may account for unexplained differences in drug response. As alleles assignment required identifying large number of variants and detection of SV, bioinformatics tool was developed in facilitate calling of star alleles from next-generation sequencing data^4^.

Current PGx resources and recommendations are based largely on a population of European descent. Studies have shown differences in pharmacogenes between ethnicities or even in closely related populations^5–8^. The aims of this study were to use WGS in investigating the prevalence of 25 high-evidence pharmacogenes (CPIC level A) star alleles and diplotypes for accurate phenotypes prediction and to examine allele frequency of known PGx variants, together with identifying potential novel deleterious variants in the Thai population.

## Results

### Star allele analysis

**Figure 1:**
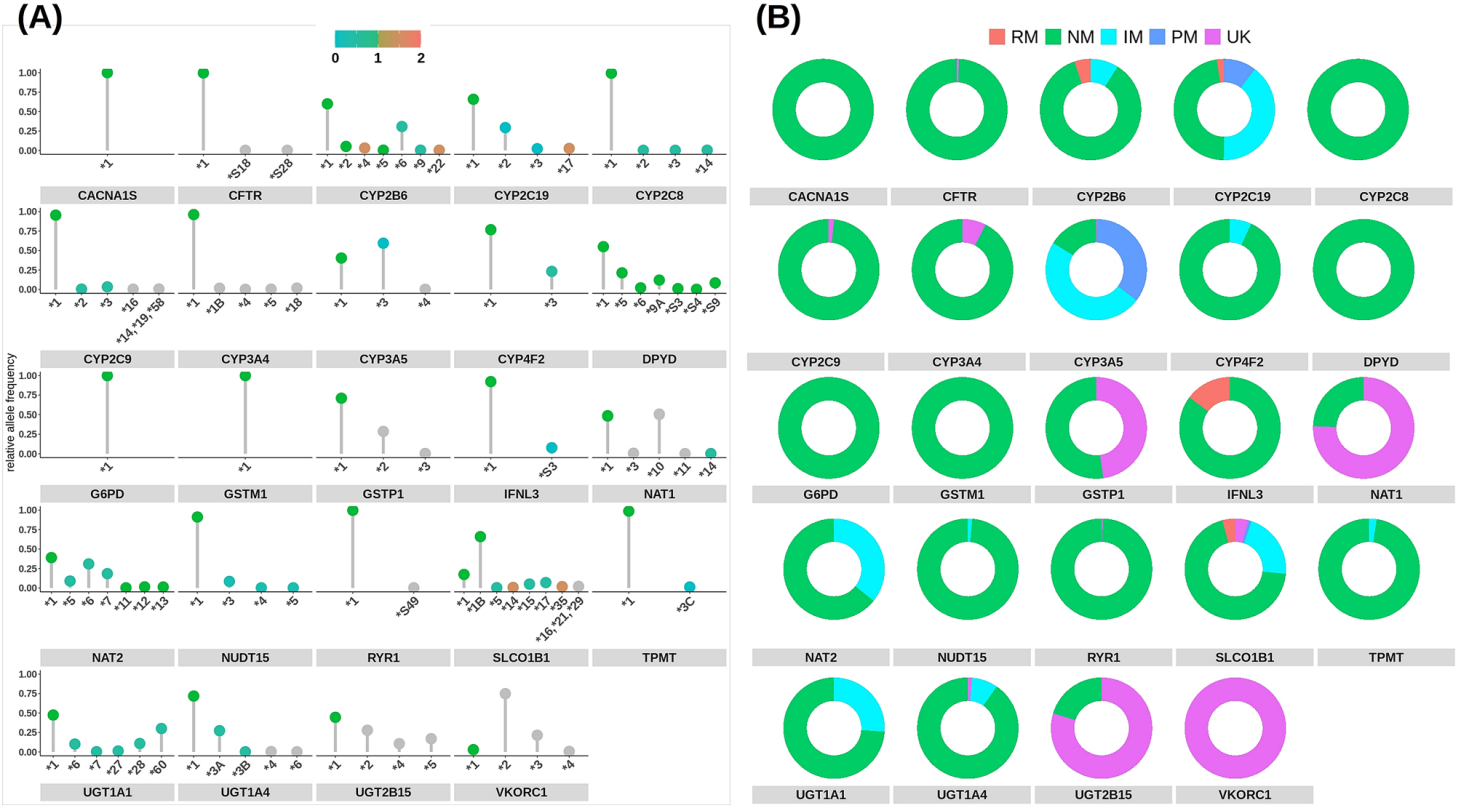
Allele frequencies of star alleles relative to alleles found within this study cohort and predicted phenotypes of 24 CPIC evidence level A pharmacogenes called using Stargazer (version 1.0.8). (**A**) Colors on dots represent activity score ranging from blue (no function) to green (normal function) to red (increased function) and grey (unknown function). (**B**) Predicted phenotypes are presented as rapid metabolizer (RM) or unfavorable response for *IFNL3*, normal metabolizer (NM) or favorable response for *IFNL3*, intermediate metabolizer (IM), poor metabolizer (PM), and unknown function (UK).

Over 25% of Thais carried high-risk diplotypes in 5 pharmacogenes including *CYP3A5*, *CYP2C19*, *NAT2*, *SLCO1B1,* and *UGT1A1* (Fig. 1). CYP3A5*3, loss-of-function allele, was found in heterozygous intermediate metabolizing (IM) diplotype, CYP3A5*1/*3 (48.5%)*,* and homozygous poor metabolizing (PM) diplotypes, CYP3A5*3/*3 (35.1%). *CYP2C19* loss-of-function *2 and *3 alleles contributed to the prevalence of IM diplotype, CYP2C19*1/*2 (36%) and *1/*3 (3%), and PM diplotype, CYP2C19*2/*2 (10%) and *3/*3 (1%). *CYP2C19* gain-of-function *17 allele was found in rapid metabolizing diplotype, CYP2C19*1/*17 (2.41%). *NAT2* slow acetylator *5, *6, and *7 alleles were found in IM diplotypes, NAT2*6/*7 (5.5%), *6/*6 (5.2%), and *5/*6 (3.1%). SLCO1B1*1B/*17, *1B/*15, and *1/*17, which are the most prevalent diplotypes that carried decreased function *5, *15, and *17 alleles, were observed at 3.95%, 3.26%, and 1.72%, respectively. UGT1A1*60/*60, *6/*60, and *28/*60 were among the most prevalent diplotypes at 10.3%, 6.29%, and 5.14%, respectively.

On the other hand, high-risk diplotype frequencies were < 3% in 10 pharmacogenes, which were *DYPD*, *CYP2C8*, *CACNA1S*, *RYR1*, *CFTR*, *NUDT15*, *CYP2C9*, *GTSM1*, *G6DP,* and *TPMT* (Fig. 1). Additionally, the functional effect of over 25% of detected alleles in *GSTP1*, *NAT1*, *UGT2B15,* and *VKORC1* was currently unknown (Fig. 1).

**Figure 2:**
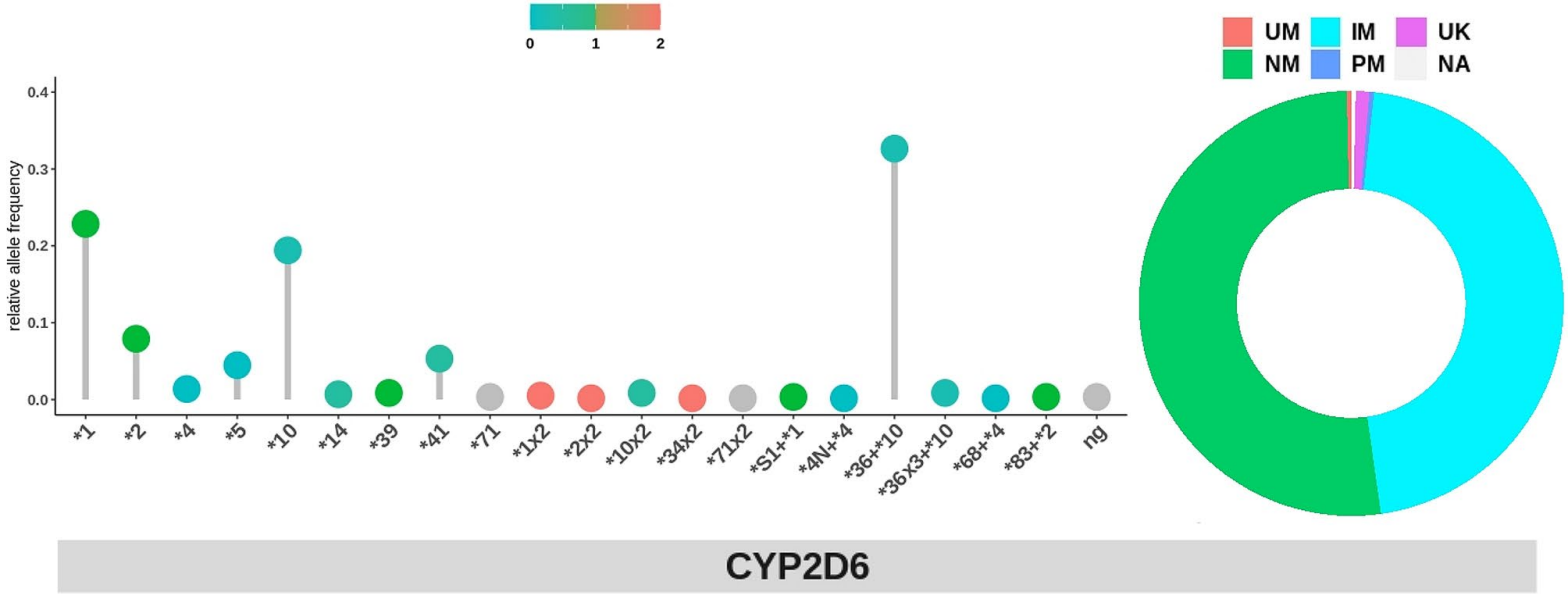
Allele frequencies of star alleles with structural variation relative to *CYP2D6* alleles found within this study cohort and predicted phenotypes called using Stargazer (version 1.0.8). (**A**) Colors on dots in star alleles plot represent activity score range from blue (no function) to green (normal function) to red (increased function) and grey (unknown function). Uncalled sample is denoted as ng. (**B**) Predicted phenotypes are presented as ultra-rapid metabolizer (UM), normal metabolizer (NM), intermediate metabolizer (IM), poor metabolizer (PM), unknown function (UK), and not applicable (NA).

Twenty different star alleles of *CYP2D6* were observed. Among these, 5 were duplication (CYP2D6*1 × 2, CYP2D6*2 × 2, CYP2D6*10 × 2, CYP2D6*34 × 2, CYP2D6*71 × *2)*, 1 was deletion (CYP2D6*5), and 6 were rearrangement (CYP2D6*S1 + *1, *4N + *4, *36 + *10, *36 × *3* + *10, *68 + *4, *83 + *2), which accounted for 1.9%, 4.5%, and 34.7% of star alleles found, respectively. CYP2D6*36 + *10 and *10 alleles were the most prevalent of *CYP2D6* decreased function alleles in this cohort. CYP2D6*1/*36 + *10, *36 + *10/*36 + *10, *10/*36 + *10, and *1/*10 were among the highest diplotypes found at 14.5%, 12.1%, 11.4%, and 9.31%, respectively (Fig. 2). Star alleles of frequencies of *CYP2D6* called through Stargazer algorithm, were within the range of the previously published East-Asian allele frequencies (Supplementary Table S4)^9^.

### Variant analysis of 25 pharmacogenes

A total of 18,825 variants were detected within 25 pharmacogenes of 291 individuals. Of 18,825 variants, 12,026 (63.8%) were rare, while 5766 (30.6%) variants were absent from the gnomAD database. An enrichment of rare variants was found within variants that impact protein function. For example, all of in-frame insertion, deletion and stop gained variants found were rare on gnomAD database in compare to 60.5% of synonymous variants and 63.5% of intron variants found were rare (Supplementary Fig. S2A). *IFNL3*, *UGT1A4,* and *CYP2D6* reported the highest number of missense variants per coding sequence, while *GSTM1*, where null genotype link to development of cancers^4^, was the most conserved (Supplementary Fig. S2B).

### Known PGx variants

**Figure 3:**
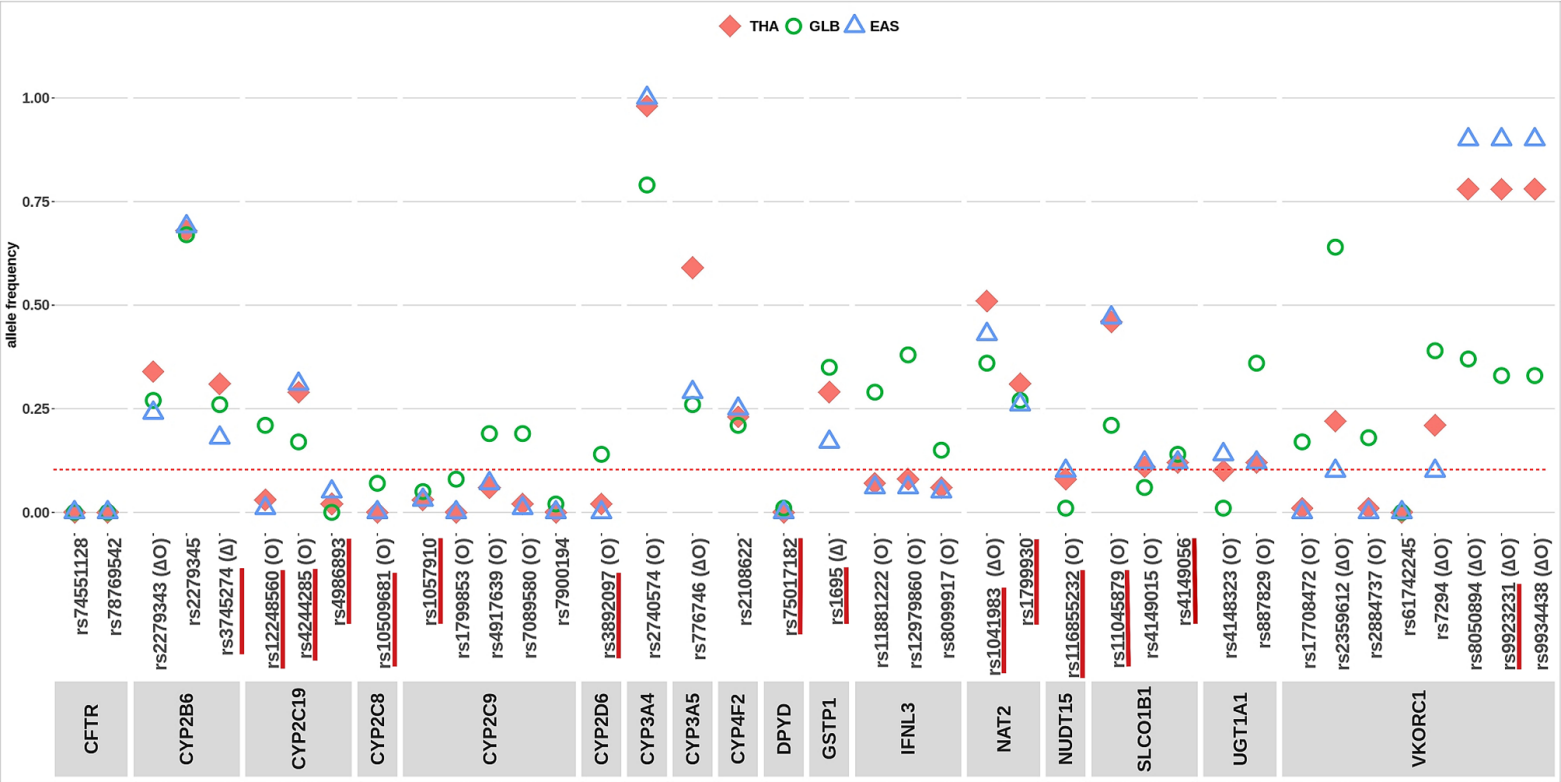
Allele frequencies of 39 high-evidence PGx variants in Thai (THA) compared to East-Asian (EAS) and global population (GLB) in gnomAD database. Variants associated with toxicity are underlined in red. Variants with significant *p*-value (*p* < 0.001) when comparing Thai allele frequency with gnomAD database, are denoted as (O), and when comparing Thai allele frequency with East-Asian population in gnomAD database, are denoted as (∆).

The prevalence of 39 high-evidence PGx variants found in Thais compared to East-Asian and global population in gnomAD database were shown in Fig. 3. Of these, 19 high-evidence PGx variants were commonly found in Thais, with allele frequency of over 0.1. Fifteen variants were associated with increased risk of toxicity or adverse drug reactions are underlined in Fig. 3^1^. Six variants were associated with increased risk of toxicity were commonly found in Thais, including rs1041983 (*NAT2*), rs1799930 (*NAT2*), rs4244285 (*CYP2C19*), rs1695 (*GSTP1*), rs4149056 (*SLCO1B1*), and rs11045879 (*SLCO1B1*). Fifty-one percent of Thais were carriers of T allele in rs1041983 (*NAT2* c.282C>T), which is associated with increased risk of liver toxicity upon treatment of anti-tuberculosis drugs^10,11^. Among the highest evidence level variants (1A), 49% of Thais carried A allele in rs4244285 (*CYP2C19* c.681G>A), which is associated with an increased risk for secondary cardiovascular events upon clopidogrel usage, and 24% of Thais carried C allele in rs4149056 (*SLCO1B1* c.521T>C), which is associated with an increased risk of simvastatin-induced myopathy^12,13^. In comparison to other populations, 26 and 10 variants were significantly different from the global and East-Asian population, respectively (Fig. 3). The rs776746 (*CYP3A5*), rs1041983 (*NAT2*), and rs2279343 (*CYP2B6*) were more frequent in Thais than both populations. Multiple variants within *VKORC1* in this cohort exhibited a significant degree of deviation from both populations.

### Potentially deleterious PGx variants

Of 305 missense variants found in this cohort, 41 variants were previously reported to associate with drug response in PharmGKB database. Novel potentially deleterious missense variants found in Thais were reported in the Supplementary Table S2.

One hundred and ten missense variants were considered novel, potentially deleterious, while 5 variants obtained Combined Annotation Dependent Depletion (CADD) PHRED-normalized scores of > 30 (Supplementary Table S2). Seventy-eight percent (n = 86) of novel potentially deleterious missense variants were only found once in this cohort. Ninety-four percent (n = 103) were rare in gnomAD database, while 61% (n = 67) were absent. Thirty percent (n = 33) had not been reported in dbSNP 150 database. Sixty-two percent of Thais carry up to 4 novel potentially deleterious missense variants.

Eleven high-confidence loss-of-function variants were found in 9 pharmacogenes (Supplementary Table S3), 8 variants were only found once in this cohort, 2 variants were rare (minor allele frequency [MAF] < 0.01), and 1 variant was found at low frequency (0.01 < MAF < 0.05). According to the gnomAD database, all loss-of-function variants were rare and 6 variants were absent. An enrichment of splice acceptor variant rs373134805 (*CYP3A5*) was found within the South East-Asian population in GenomeAsia 100 k database^14^.

## Discussion

This is the first study to report the prevalence of star alleles, diplotypes, and phenotype predictions of 25 clinically relevant pharmacogenes, including *CYP2D6* SV, from WGS in the Thai population. We then identified 39 known PGx variants in Thais and compared allele frequencies to East-Asian and global populations. We further identified 110 novel potentially deleterious missense variants and 11 high-confidence loss-of-function variants circulating within the population.

The “star” nomenclature system used in this study is a powerful tool for predicting activity or function of enzymes, transporters, or drug targets, as it accounts for a combination effect of multiple variants within an allele^15^. We found high clinical relevance cytochrome P450 genes (*CYP3A5, CYP2C19*, and *CYP2D6*) exhibiting high variation in predicted phenotype. This could reflect the low evolutionary constraint within these enzymes, as they lack essential endogenous function^16^. SV, between *CYP2D6* and its pseudogenes (*CYP2D7, CYP2D8*) established to alter enzymatic activity, found to exerted high importance in the Thai population^17^. It accounted for 60% of *CYP2D6* star alleles detected and 83.8% of all high-risk diplotypes in this study. Interestingly, prevalence of *CYP2D6* SV was also previously reported to be highest among Asians when compared to African Americans, Caucasians, and Hispanics^17^. Our finding emphasizes the importance of detecting *CYP2D6* SV for accurate phenotype prediction especially in Thai population.

Variability in drug response among ethnicities had long been observed, but a recent increase in the number of populations studied unveiled another layer of genetic variability within the sub-population, such as distribution gradient of CYP2C19*17 found from Western to Eastern Europe^5^. In Thais, CYP3A5*3 (rs776746), CYP2B6*6 (rs2279343), and *NAT2* (rs1041983) were significantly higher compared with East-Asian and global populations. Varying allele frequency of multiple *VKORC1* variants to different populations found in this study supported the previously reported variation of rs9923231 among the East-Asian population where allele frequency of 0.96, 0.94, and 0.90 was observed in North-East Asians (Japanese, South Koreans, and Chinese) in comparison to 0.62, 0.69, 0.75 observed in South-East Asians (Filipinos, Malaysians, and Indonesians)^14^. In addition to known PGx variants, novel potentially deleterious variants were population specific with 94.2% identified were rare in gnomAD database, and 60.3% were absent. This reflect previous finding that high impact variants are often rare and geographically localized as a result of purifying selection^18^. For example, potentially deleterious splice acceptor variant c.433-1G>C in *CYP3A5* found at a low frequency in Thai (0.017) is population-specific South East-Asian populations including Vietnamese (0.018), Malaysian (0.039), and Indonesian (0.015), while extremely rare in the gnomAD database^14^. These variations within subpopulations of East-Asians demonstrate the benefit of PGx testing and highlight the precaution that must be taken when associating PGx with ethnicity labels.

A focus on rare variants in explaining inter-individual variation in drug response is likely to increase as the cost of sequencing is reduced, making WGS more readily available. An important challenge remains in interpreting these rare variants of unknown significant. Repository SPHINX (Sequence, Phenotype, and pHarmacogenomics INtegration eXchange https://emergesphinx.org), that link PGx variants of unknown significance with patients clinical phenotypes would facilitate researchers on studying these variants of unknown significant for future PGx discovery^19^.

The WGS ability to access PGx variants and SV in a single methodology reduced time and labor involved. This study demonstrates WGS to be a highly efficient platform in research and PGx testing. The current high cost and bioinfomatics required to process and translate large data could limit WGS application as a PGx testing platform in routine clinical setting. Development of bioinformatics tools use in translating genotype data are moving toward a more automated manner, such as under developing PharmCAT^20^. This would make interpreting WGS data more user-friendly and accessible to wider healthcare provider in the near future. In the meantime, an alternative more cost effective platform such as genotyping arrays could currently be a more applicable^21^.

We acknowledge several limitations and ways the study could be improved. A portion of enrolled participants was Brugada syndrome patients. Although none of the genes associated with Brugada syndrome were examined, results could be enhanced with unknown genetic factors influencing the disease. Previous study reported an inconsistent in star alleles calling in samples with complex SV when three bioinformatics tools were compared, this suggest that further confirmation, such as using high-resolution long-read sequencing that allows accurate variant calling and phasing, might be required in some samples with *CYP2D6* complex SV^22^. Computational prediction tools like Loss-of-Function Transcript Effect Estimator (LOFTEE) and Combinded Annotation Dependent Depletion (CADD) used in this study and other studies are useful in prioritizing deleterious effects in variants of unknown significance; however, variants must be reported with caution and validated through a functional study before implementation in clinical settings^23,24^.

In conclusion, we reported a comprehensive overview of the PGx spectrum in a Thai population and its differences with East-Asian populations. We demonstrated the utilization of WGS in PGx testing, including accurate phenotype prediction using the “star” nomenclature system, SV detection, and identification of known and unknown potentially deleterious PGx variants. The reported findings and variations within pharmacogenes of the Thai population facilitate PGx-guided clinical decision making in Thailand and contribute to the database of the understudied South-East Asian population for further application of precision public health including dosing guidelines, drug development, clinical trials, and development of population-specific screening.

## Materials and methods

WGS of 291 individuals from the Brugada cohort (Clinical Trial Registration Number NCT04232787), consisting of 108 patients with Brugada syndrome and 183 controls were enrolled into the study. Controls had no type I Brugada pattern on standard 12-lead echocardiogram or family history of sudden cardiac arrest and were matched to the cases for place of birth. Controls were volunteers from blood donor at multiple sites of National Blood Center, Thai Red Cross Society (n = 99) or visitors for health check-up and workers at King Chulalongkorn Memorial Hospital (n = 84). All subjects, case and control, were of Thai ethnic origin by self-report from 52 provinces in Thailand which can be separated into 5 major geographical regions: north, northeast, central, east and south as shown in Supplementary Table S1. The study was approved by the Institutional Review Board of the Faculty of Medicine, Chulalongkorn University, Bangkok, Thailand (IRB No. 431/58). Informed consent was obtained from all participants. All methods were performed in accordance with relevant guidelines/regulations.

Sequencing of paired-end 150 bp fragment reads from polymerase chain reaction (PCR)-free sequencing libraries was performed on the HiSeqX (Illumina Ltd, Cambridge, UK). Sequencing, alignment, and variant calling were performed at Illumina Ltd, Cambridge, UK. Reads were aligned to NCBI GRCh38 human reference genome assembly. All samples have minimum mean depth coverage of 30 × with minimum of 97% autosome coverage at 10 × and 94% aligned reads.

All variants within the region specified in Stargazer (version 1.0.8) for 25 pharmacogenes, including *CACNA1S*, *CFTR*, *CYP2B6*, *CYP2C8*, *CYP2C9*, *CYP2C19*, *CYP2D6*, *CYP3A4*, *CYP3A5*, *CYP4F2*, *DPYD*, *G6PD*, *GSTM1*, *GSTP1*, *IFNL3*, *NAT1*, *NAT2*, *NUDT15*, *RYR1*, *SLCO1B1*, *TPMT*, *UGT1A1*, *UGT1A4*, *UGT2B15,* and *VKORC1*, were extracted from genome Variant Call Format (VCF) files using BCFtools (version 1.10.2). Variants were excluded if they were with locus GQX < 30, with site genotype conflicted with proximal indel call, with locus in the region with conflicting indel calls, and with unbalanced phasing pattern. VCF files of all samples were merged to generate a single VCF file. Non-variants were later excluded from the final VCF file.

Multidimensional scaling analysis was performed on 15,965 single nucleotide polymorphism, excluding indels, within 25 pharmacogenes using Plink (version 1.9). Multidimensional scaling plot illustrate no separation between cases and control for the first 4 components (Supplementary Fig. S1).

### Star allele analysis

Stargazer required a VCF file on genome coordinate GRCh37 and a gdf file for SV detection. Genome coordinates, reference, and alternative allele were converted from GRCh38 to GRCh37 using LiftoverVariants tools available in GATK package (version 4.1.6.0). To generate the gdf file for SV detection of *CYP2D6*, first, Bazam (version 1.0.1) was used to extract *CYP2D6*-*CYP2D7* region from BAM file and realigned on GRCh37 coordinates^25^. Samtools (version 1.9) was used to extract read depth. Sdf2gdf script, which was available on Stargazer, was used to generate the gdf files. One sample with high missingness within the *CYP2D6* region was excluded when calling star allele of *CYP2D6.* The haplotype, activity score, diplotype, and predicted phenotype called by Stargazer with *VDR* as a control gene were combined and visualized using R program (version 3.6.3, dplyr and ggplot2 package). High-risk diplotypes were defined as diplotypes which predicted phenotype was not normal.

### Variant analysis of pharmacogenes

The VCF file was annotated with gnomAD allele frequencies of the global population using Ensemble Variant Effect Predictor (version 98.3)^26^. Annotated variants were classified into common (MAF ≥ 0.05), low frequency (0.05 > MAF ≥ 0.01), rare (MAF < 0.01), and absent (MAF = 0). Variants within each group were counted using VCFtools (version 0.1.15). Number of missense variants per coding sequence was calculated by $\frac{\text{number \,of \,missense \,variants}}{\text{Ensembl\, transcript\, length}}$, where Ensembl transcript length was obtained from BioMart database (https://www.Ensembl.org/biomart/martview/) and had APPRIS annotation value as “primary assembly.” PGx variants were retrieved from PharmGKB database (https://www.pharmgkb.org, accessed on 06/06/2020). Variants with evidence level 1A, 1B, and 2A were identified as known PGx variants. Allele frequencies were compared with those of the population in gnomAD using Chi-square test or Fisher’s exact test. A p-value of < 0.001 was used as a significant level after Bonferroni correction.

Missense variants were extracted to examine novel potentially deleterious variants where variants reported in PharmGKB database or used in star allele analysis were excluded. CADD PHRED-normalized scores were obtained online. CADD PHRED-normalized scores ≥ 20, or 1% most deleterious single nucleotide variants within the reference genome, were considered potentially deleterious variants. Loss-of-function variants include stop-gained, splice-site disrupting, frameshift insertion, and frameshift deletion variants. LOFTEE algorithm available in VEP-plugin was used to determine loss-of-function variants, and variants annotated as “high confidence” were considered loss-of-function in this study.

## Supplementary information

Supplementary Information.

## Data Availability

The data that support the findings of this study are available from the National Research Council of Thailand but restrictions apply to the availability of these data, which were used under license for the current study, and so are not publicly available. Data are; however, available from the authors upon a reasonable request and with a permission of the National Research Council of Thailand.
