# Supplementary material for: Phenotype prediction and characterization of 25 pharmacogenes in Thais from whole genome sequencing for clinical implementation
Source: Sci Rep. 2020 Nov 3;10:18969. doi: 10.1038/s41598-020-76085-3 (PMC7641128; doi:10.1038/s41598-020-76085-3)
Supplement: Supplementary file 1 — Supplementary Information. [file 41598_2020_76085_MOESM1_ESM.pdf]

Phenotype Prediction and Characterization of 25 Pharmacogenes in Thais from Whole Genome Sequencing for Clinical Implementation

John Mauleekoonphairoj[1,2], Monpat Chamnanphon[3,4], Apichai Kongpattanayothin[1,5,6], Boosamas Sutjaporn[1], Pharawee Wandee[1], Yong Poovorawan[7], Koonlawee Nademanee[1,8,9], Monnat Pongpanich[10,11], Pajaree Chariyavilaskul[3,4,*]

[1]Center of Excellence in Arrhythmia Research Chulalongkorn University, Department of Medicine, Faculty of Medicine, Chulalongkorn University, Bangkok, Thailand

[2]Interdisciplinary Program of Biomedical Sciences, Graduate School, Chulalongkorn University, Bangkok, Thailand

[3]Clinical Pharmacokinetics and Pharmacogenomics Research Unit, Faculty of Medicine, Chulalongkorn University, Bangkok, Thailand

[4]Department of Pharmacology, Faculty of Medicine, Chulalongkorn University, Bangkok, Thailand

[5]Division of Cardiology, Department of Pediatrics, Faculty of Medicine, Chulalongkorn University, Bangkok, Thailand

[6]Bangkok General Hospital, Bangkok, Thailand

[7]Department of Pediatrics, Faculty of Medicine, Chulalongkorn University, Bangkok, Thailand,

[8]Department of Medicine, Faculty of Medicine, Chulalongkorn University, Bangkok, Thailand

[9]Pacific Rim Electrophysiology Research Institute, Bumrungrad Hospital, Bangkok, Thailand

[10]Department of Mathematics and Computer Science, Faculty of Science, Chulalongkorn University, Bangkok, Thailand

[11]Omics Sciences and Bioinfomatics Center, Faculty of Science, Chulalongkorn University, Bangkok, Thailand

*Correspondence to: Pajaree Chariyavilaskul, MD, PhD.

Department of Pharmacology, Faculty of Medicine,

Chulalongkorn University, Bangkok, Thailand

Email: pajaree.l@chula.ac.th

Tel: +66816134664, +6622564481 ext. 3020

Supplementary Information

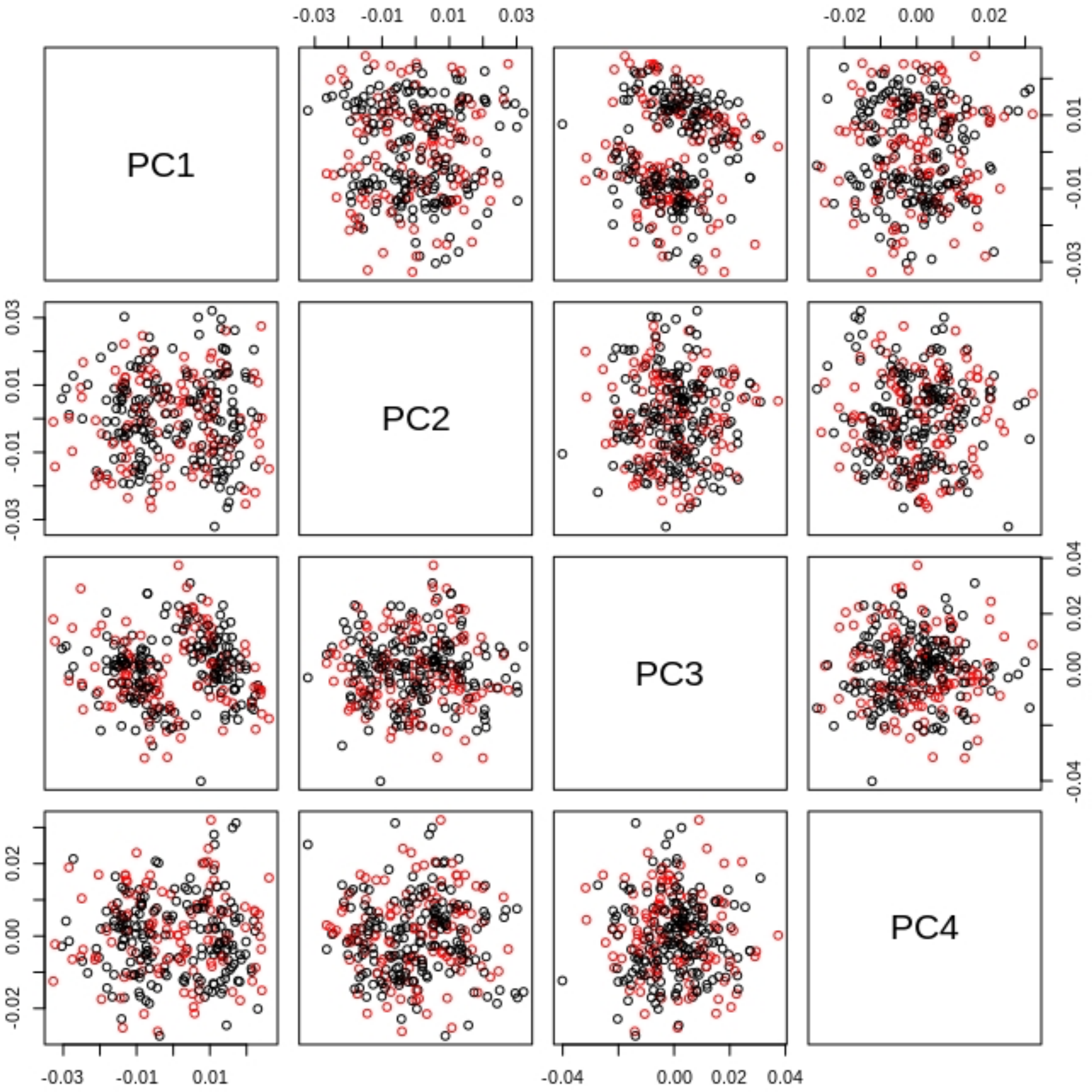

Supplementary Figure S1. Multidimensional scaling plot of the first 4 components conducted with 15,965 single nucleotide polymorphisms within 25 pharmacogenes of 108 cases (red) and 183 control (red) using Plink (version 1.9).

(A)

(B)

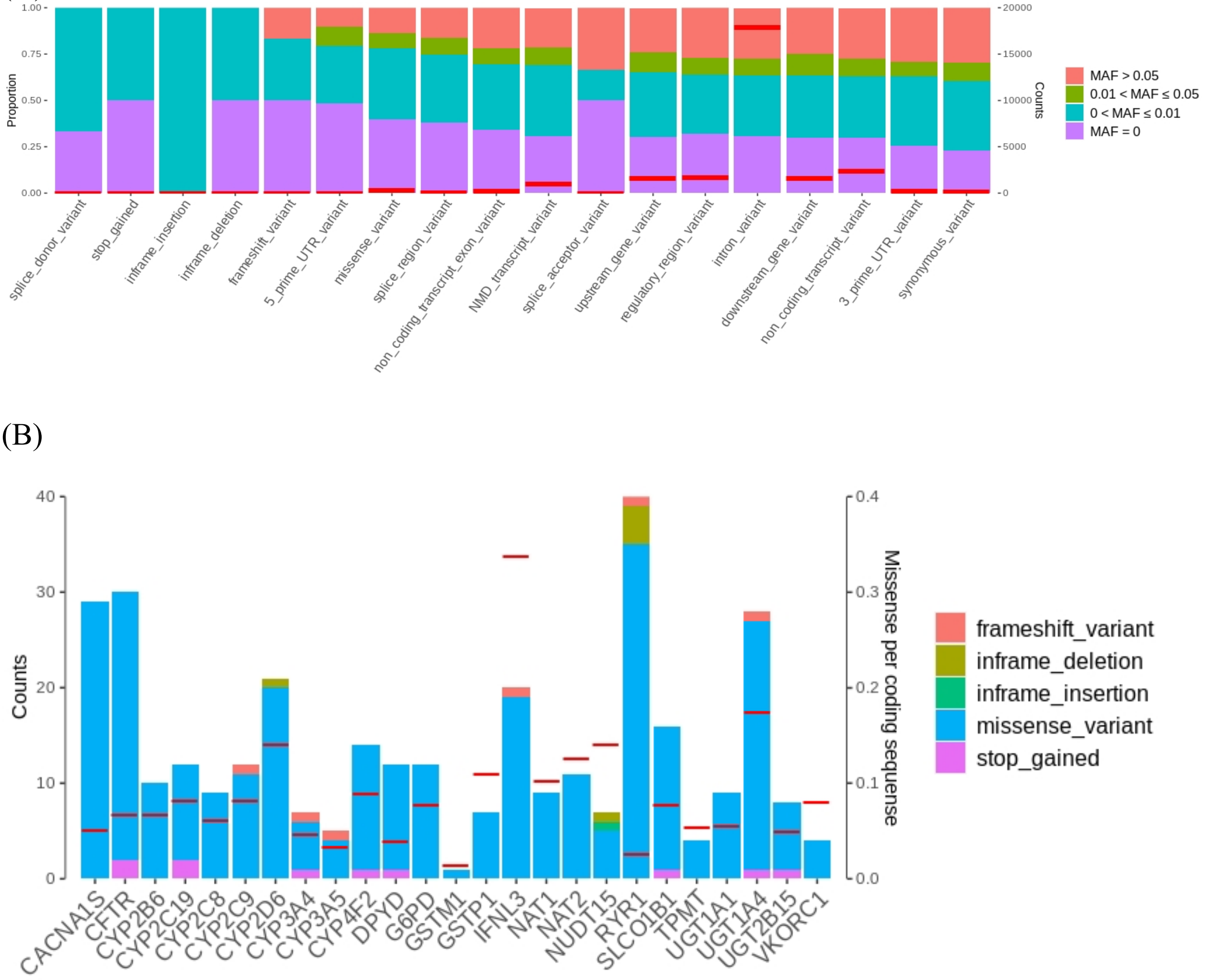

Supplementary Figure S2. Distribution of variants found within 25 pharmacogenes. (A) Proportion of variants grouped by allele frequency relative to gnomAD database and number of variants found within each type of variant show in red dash (-). (B) Counts of variant that impact protein function within each gene and missense variant per coding sequence per gene show in red dash (-).

Supplementary Table S1. Geographical regions and allele frequencies of 39 known PGx variants of case and control samples.

		Case (n=108)	Control (n=183)
**North**		17	37
**Northeast**		41	61
**Central**		37	66
**East**		4	6
**South**		4	6
**Allele frequency**	rs75017182	0.00	0.01
	rs887829	0.13	0.11
	rs4148323	0.08	0.11
	rs776746	0.67	0.55
	rs2740574	0.98	0.99
	rs74551128	0.00	0.00
	rs78769542	0.00	0.00
	rs1041983	0.47	0.52
	rs1799930	0.29	0.32
	rs12248560	0.03	0.02
	rs4986893	0.02	0.02
	rs4244285	0.31	0.28
	rs1799853	0.00	0.00
	rs7900194	0.00	0.00
	rs7089580	0.02	0.02
	rs4917639	0.06	0.06
	rs1057910	0.04	0.03
	rs10509681	0.00	0.00
	rs1695	0.29	0.29
	rs4149015	0.09	0.11
	rs4149056	0.13	0.12
	rs11045879	0.48	0.45
	rs116855232	0.08	0.08
	rs7294	0.22	0.21
	rs2359612	0.23	0.23
	rs8050894	0.77	0.78
	rs9934438	0.77	0.78
	rs17708472	0.01	0.01
	rs2884737	0.01	0.00
	rs61742245	0.00	0.00
	rs9923231	0.77	0.78
	rs2108622	0.19	0.26
	rs11881222	0.07	0.08
	rs12979860	0.07	0.08
	rs8099917	0.05	0.06
	rs3745274	0.33	0.30
	rs2279343	0.36	0.33
	rs2279345	0.69	0.67
	rs3892097	0.02	0.02

Supplementary Table S2. Novel potentially deleterious pharmacogenomics variants; **Ref/Alt:** Reference/Alternative nucleotide; **Ref_AA:** Reference Amino Acid; **Alt_AA:** Alternative Amino Acid; **CADD score:** Combined Annotation Dependent Depletion PHRED-normalized scores.

Position (GRCh38)	dbSNP150	Ref/Alt	Allele Count	Gene	Ref_AA	Alt_AA	CADD score
**chr1:201062468**	.	T/C	1	CACNA1S	E	G	33
**chr7:117614633**	rs1005269197	G/A	1	CFTR	G	S	32
**chr1:201078026**	rs780841536	T/C	1	CACNA1S	Y	C	31
**chr19:38452983**	.	T/G	1	RYR1	L	R	31
**chr1:201048591**	rs745558537	T/G	1	CACNA1S	K	Q	30
**chr19:38516212**	rs878984852	G/A	1	RYR1	R	Q	29.8
**chr22:42127527**	rs769351604	G/A	1	CYP2D6	R	C	29.2
**chr7:117592595**	rs377447726	A/G	1	CFTR	R	G	28.7
**chr1:201083251**	.	T/G	2	CACNA1S	Y	S	28.5
**chr1:201078005**	rs150590855	C/A	1	CACNA1S	R	L	28.5
**chr1:201052633**	rs555016254	C/T	1	CACNA1S	A	T	28
**chr1:201061410**	.	G/A	1	CACNA1S	R	C	27.4
**chr1:97305348**	rs570122671	G/A	1	DPYD	T	I	27.3
**chr6:18133821**	rs777803269	T/G	1	TPMT	D	A	27.2
**chr7:117504290**	rs1800073	C/T	1	CFTR	R	C	27.1
**chr7:117504306**	.	A/C	2	CFTR	D	A	26.9
**chr1:201047168**	rs3850625	G/A	19	CACNA1S	R	C	26.8
**chr1:201060666**	rs145039828	C/T	1	CACNA1S	G	S	26.6
**chr19:38536011**	.	A/G	1	RYR1	N	S	26.6
**chr7:117587821**	.	T/C	1	CFTR	I	T	26.6
**chr1:201065924**	rs571902899	C/T	1	CACNA1S	V	M	26.5
**chr1:201077922**	rs557195329	C/T	1	CACNA1S	V	M	26.4
**chr16:31094573**	rs781304132	G/T	1	VKORC1	R	S	26.2
**chr11:67584499**	rs755557033	C/G	1	GSTP1	Q	E	26
**chr19:15879844**	rs372871763	C/T	1	CYP4F2	R	Q	26
**chr19:38444648**	.	T/C	1	RYR1	M	T	26
**chr19:38512443**	.	C/G	1	RYR1	F	L	25.9
**chr2:233772309**	rs114982090	C/T	5	UGT1A8	P	L	25.9
**chr1:201043401**	.	A/G	1	CACNA1S	F	S	25.7
**chr1:201083173**	rs143202536	G/T	1	CACNA1S	T	N	25.7
**chr12:21224811**	rs377350683	T/C	1	SLCO1B1	C	R	25.7
**chr19:38502914**	.	C/G	1	RYR1	R	G	25.7
**chr19:39243685**	rs77379751	G/A	31	IFNL3	R	C	25.6
**chr19:38519384**	rs201339536	G/A	2	RYR1	E	K	25.6
**chr1:201089374**	rs186538122	G/A	1	CACNA1S	R	W	25.5
**chr19:38519282**	rs775895899	G/A	1	RYR1	G	R	25.5
**chr19:38499811**	rs575780192	C/T	1	RYR1	R	W	25.4
**chr19:15892373**	rs754089074	G/A	2	CYP4F2	A	V	25.3
**chr1:201070353**	.	G/A	1	CACNA1S	P	L	25
**chr1:201040054**	rs12139527	A/G	68	CACNA1S	L	S	24.9
**chr13:48041009**	rs773719265	C/A	1	NUDT15	S	Y	24.9
**chrX:154535348**	rs886044847	A/G	1	G6PD	F	S	24.8
**chr6:18147901**	rs752440908	T/C	1	TPMT	H	R	24.6
**chr7:117540282**	rs1800086	C/G	1	CFTR	T	S	24.6
**chr12:21178618**	.	T/A	1	SLCO1B1	F	Y	24.5
**chr19:38565511**	.	G/A	2	RYR1	G	S	24.5
**chr1:201047143**	.	C/T	1	CACNA1S	R	Q	24.4
**chr1:201110216**	rs12406479	G/C	1	CACNA1S	A	G	24.4
**chr19:15886018**	rs145174239	G/C	1	CYP4F2	L	V	24.3
**chr1:201076930**	rs142356235	C/T	1	CACNA1S	S	N	24.2
**chr19:38502628**	rs754579512	T/G	1	RYR1	V	G	24.2
**chr19:38448375**	rs368711923	G/A	1	RYR1	R	H	24.1
**chr8:18222050**	.	G/A	1	NAT1	M	I	24.1
**chr19:38505076**	rs566495420	G/A	3	RYR1	D	N	24
**chr7:117592588**	rs1800103	A/G	1	CFTR	I	M	24
**chr8:18222649**	rs768813958	A/T	3	NAT1	D	V	24
**chr11:67584472**	rs774305853	G/A	1	GSTP1	A	T	23.8
**chr7:117535318**	rs121909046	A/G	2	CFTR	E	G	23.8
**chr8:18400392**	.	A/C	1	NAT2	Q	P	23.8
**chr19:41006980**	.	G/T	1	CYP2B6	R	L	23.7
**chr19:41012471**	rs201500445	T/C	3	CYP2B6	Y	H	23.7

**chr19:38565443**	.	G/A	1	RYR1	G	D	23.7
**chr4:68663024**	.	G/T	1	UGT2B15	A	D	23.7
**chr7:117530977**	.	T/C	1	CFTR	S	P	23.7
**chr19:39243850**	rs139076671	G/A	1	IFNL3	H	Y	23.6
**chr8:18222271**	.	T/C	1	NAT1	L	P	23.6
**chr7:117531043**	rs145900055	C/T	1	CFTR	P	S	23.5
**chr1:201089385**	rs35534614	C/T	1	CACNA1S	G	D	23.4
**chr19:15878779**	rs3093200	G/T	3	CYP4F2	L	M	23.4
**chr19:38469044**	rs780626994	C/T	1	RYR1	L	F	23.4
**chr4:68668066**	rs192628779	A/G	5	UGT2B15	C	R	23.4
**chr1:201051079**	.	G/A	1	CACNA1S	P	S	23.3
**chr19:39244019**	rs149832972	G/A	1	IFNL3	L	F	23.3
**chr19:38504293**	.	C/T	1	RYR1	T	I	23.3
**chr4:68654253**	rs187815441	T/C	1	UGT2B15	H	R	23.3
**chr7:117592287**	.	C/G	1	CFTR	S	C	23.3
**chr7:117627561**	.	C/T	2	CFTR	P	S	23.3
**chr10:94781959**	rs764137538	C/T	1	CYP2C19	R	W	23.2
**chr7:117559577**	.	T/G	1	CFTR	I	M	23.2
**chr19:39244114**	rs145428712	G/A	1	IFNL3	T	M	23.1
**chr19:38570667**	.	A/G	1	RYR1	I	V	23.1
**chr19:38485972**	rs192863857	C/T	4	RYR1	P	S	23.1
**chr10:94775447**	rs150152656	C/T	1	CYP2C19	T	M	22.9
**chr2:233772416**	rs371183955	C/T	4	UGT1A9	H	Y	22.9
**chr10:94947843**	.	T/G	1	CYP2C9	I	M	22.8
**chr2:233718944**	rs553189135	C/A	1	UGT1A4	L	I	22.8
**chr4:68670516**	rs529876617	G/T	1	UGT2B15	H	N	22.8
**chr8:18400653**	rs568110818	T/A	1	NAT2	F	Y	22.8
**chr1:201083231**	rs572977674	C/T	1	CACNA1S	V	I	22.7
**chr11:67586206**	rs4986949	G/T	3	GSTP1	D	Y	22.6
**chr1:97193101**	rs766833304	G/C	1	DPYD	P	A	22.3
**chr19:38492540**	rs35364374	G/T	10	RYR1	G	C	22.3
**chr19:41004380**	rs535039125	C/T	1	CYP2B6	R	W	22.2
**chr19:38485976**	rs199837883	C/T	2	RYR1	P	L	22.2
**chr1:201110258**	rs549107212	G/A	1	CACNA1S	T	M	22
**chr12:21200625**	rs752196141	T/C	1	SLCO1B1	V	A	22
**chr13:48041096**	.	T/C	1	NUDT15	V	A	22
**chr19:38578027**	rs373919284	C/T	1	RYR1	P	L	22
**chr19:38527689**	rs538497899	C/T	3	RYR1	R	W	22
**chr1:201089392**	rs190152688	T/C	2	CACNA1S	I	V	21.8
**chr19:15892398**	rs556151888	G/A	1	CYP4F2	R	C	21.8
**chr8:18222637**	rs1044890902	G/A	1	NAT1	R	Q	21.8
**chr19:38527707**	rs55876273	G/C	3	RYR1	E	Q	21.5
**chr10:95064936**	rs750028311	A/G	1	CYP2C8	I	T	21.4
**chrX:154532206**	.	A/G	1	G6PD	I	T	21.1
**chr11:67584478**	rs12796085	C/G	1	GSTP1	L	V	21
**chr19:38565544**	.	G/C	1	RYR1	D	H	20.8
**chr7:117594979**	rs562851847	A/G	1	CFTR	N	S	20.5
**chr7:99660591**	.	T/C	1	CYP3A5	S	G	20.5
**chr8:18400082**	rs765487420	A/C	1	NAT2	I	L	20.4

Supplementary Table S3. Loss of function pharmacogenomics variants; **Ref/Alt:** Reference/Alternative nucleotide;**MAF:** Minor Allele Frequency.

**Position (GRCh38)**	**Ref/Alt**	**dbSNP150**	**GENE**	**Annotation**	**MAF in Thai**	**MAF in gnomAD**		**MAF in 100k GenomeAsia**	
						Global	EAS	NEA	SEA
**chr7:99666690**	C/G	rs373134805	CYP3A5	splice_acceptor_variant	0.017	3.18E-05	0	0	0.022
**chr10:94842889**	C/A	rs370320936	CYP2C19	stop_gained	5.15E-03	0	0	0	1.45E-03
**chr12:21224840**	G/A	rs200994482	SLCO1B1	splice_donor_variant	3.45E-03	1.60E-04	3.22E-03	0	1.45E-03
**chr7:117611708**	G/A		CFTR	stop_gained	1.75E-03	0	0	0	0
**chr7:99666950**	A/G	rs55965422	CYP3A5	splice_donor_variant	1.72E-03	4.46E-04	8.99E-03	5.70E-03	1.45E-03
**chr10:94941978**	AG/A		CYP2C9	frameshift_variant	1.72E-03	0	0	0	0
**chr1:97828127**	G/A	rs189768576	DPYD	stop_gained	1.72E-03	3.19E-05	6.41E-04	1.42E-03	0
**chr7:117559463**	G/A	rs397508200	CFTR	splice_acceptor_variant	1.72E-03	0	0	0	0
**chr7:117592292**	C/T	rs121908760	CFTR	stop_gained	1.72E-03	0	0	0	0
**chr19:15897501**	C/T	rs752022409	CYP4F2	stop_gained	1.72E-03	3.19E-05	6.42E-04	0	0
**chr19:39243908**	C/T	rs546666114	IFNL3	splice_acceptor_variant	1.72E-03	0	0	0	0

Supplementary Table S4. Percentage of *CYP2D6* star alleles in Thais and East-Asian population.

**Alleles**	**This study (%)**	**Suwannasri *et al.*, 2011[32]** (%) **(n = 288)**	**Chamnanphon *et al.*, 2013[33]** (%) **(n = 57)**	**Gaedigk *et al.*, 2017[21] East-Asian** (%) **(n = 14,816)**	
				Average	Range
***1**	22.93	22.91	35	35.24	17.5-93.79
***2**	7.93	9.7	9.6	13.11	7.65-42.71
***4**	1.38	0.7	0.9	0.59	0-4.35
***5**	4.48	4.3	4.4	5.17	0-9.6
***10**	19.48	44.6	45.6	42.58	8.6-64.1
***14**	0.69	1.04	0.9	0.77	0-3
***36**	-	16.4	0.9	1.52	0-16.4
***39**	0.86	-	-	0.24	0-1.18
***41**	5.34	-	1.8	2.18	0-6.54
***71**	0.34	-	-	0.52	0-1.5
***1x2**	0.52	-	0	0.27	0-0.51
***2x2**	0.17	-	0	0.38	0-0.99
***10x2**	0.86	-	0	0.4	0-1
***71x2**	0.17	-	0	0.03	0-0.2
**Other duplication**	0.17	0.35	-	1.39	0-6
***36+*10**	32.76	-	-	26.41	22.45-32.65
***36x3+*10**	0.86	-	-	1.02	1.02-1.02
**Other rearrangements**	1.03	-	-	5.51	5.51-5.51
